# Horse Sickness

**Published:** 1902-06

**Authors:** W. Ramsey Smith

**Affiliations:** Veterinary Surgeon, Desmond, South Australia


### HORSE SICKNESS.

By Dr. W. Ramsey Smith, B.S.C., M.B., C.M.,
VETERINARY SURGEON, DESMOND, SOUTH AUSTRALIA.

In 1854-55 Cape Colony was visited by an extensive outbreak of horse sickness that carried off 64,850 horses, mares, and foals, representing a value of over half a million sterling. There have been other epidemics at various periods from 1780 down to recent times. In 1891-92, 14,000 horses died.

The epidemic of 1854–55 was investigated by the Cape of Good Hope Agricultural Society through circulars sent out to the Civil Commissioners of the various districts of the colony. The conclusion arrived at was that horses were liable to horse sickness if they were left out in the night air in a season of general malaria and permitted to eat grass which had become dew-laden.

In 1886 the Imperial Veterinary Department sent out Mr. Joshua Nunn, a veterinary surgeon, as specially fitted for the investigation of this disease on account of his experience of diseases of horses and cattle in India. He was instructed to make a report upon the horse sickness as it appeared in various parts of South Africa. At the end of a year he reported that the horse sickness is not anthrax. After another year's work he was able to report that it is not contagious, and is due to climatic causes. So far all investigations had reached conclusions regarding what the disease was not, but there was very little information as to its true nature and pathology.

In 1891 Dr. Edington, F.R.S. (Edin.), went out from Edinburgh as Director of the Colonial Laboratory of Bacteriology. One of his first duties was to investigate the nature, origin, and occurrence of this disease, and, if possible, to discover a cure for the malady. In his early investigation he had the help of Messrs. Wiltshire and Raymond, who had formerly carried on investigations with Mr. Joshua Nunn. Later on he was assisted by Dr. Carrington Purvis and Dr. R. S. Black. Dr. Edington has reported at various times, generally on the symptoms and incidence of the disease, and especially on its pathology and bacteriology ; and for some years he has been diligently searching for a mode of successful treatment.

Lately, when in South Africa, I paid a visit to the Colonial Bacteriological Laboratory at Grahamstown, where Dr. Edington and others have been working on this subject. Mr. Desmond accompanied me, and we spent some days in examining pathological and bacteriological specimens connected with the disease and in discussing the symptoms, lines of investigations, and other problems connected with it. I had also the advantage of discussing these problems with Dr. Carrington Purvis, who, like Dr. Edington, had formerly been my colleague as assistant professor at Edinburgh University. I found he had been carrying out investigations into certain diseases in South Africa on lines he had laid down and discussed with me more than ten years ago. Mr. Desmond and I were thus in a position to know what facts were settled regarding this sickness, what points were in dispute, and what problems were under investigation. We also spent some time at the Imperial Remount Depot at Port Elizabeth, which was under the care of several eminent veterinary surgeons, and we were for two or three weeks in company with Captain Hayes, F.R.C.V.S., a writer whose veterinary reputation is world-wide, and an enthusiast in everything pertaining to horses and veterinary pathology. On a former visit to South Africa, in 1892 he had seen cases and made inquiries regarding the disease in Cape Colony, Orange River State, the Transvaal, and Natal.

Hitherto this particular lung sickness of horses had not been found outside of Africa. It is even doubtful if outbreaks that have been reported from North and Central Africa are identical with or even similar to the sickness as manifested in Cape Colony, the Transvaal, Natal, and other parts of South Africa.

Three forms of the disease are recognized. First—*dun-paardzietke*, horse sickness ; second, *dikkop-zietke*, or swelled head ; third, *blutong*, so called on account of the bluish color of the tongue. For a time it was thought that the disease was an uncommonly rapid one, on account of the victims, apparently well, becoming all at once acutely ill, and dying with well-marked symptoms within a few days or even hours. For some time it was found impossible to transmit the disease directly from one animal to another, but this has been successfully accomplished by Dr. Edington in two different ways ; and, as a result of this, it has been established that the disease in its acute form has a definite incubation period of from eight to nine days.

The symptoms during life are not absolutely characteristic of this disease, since most of them, if regarded singly, might be ascribed to other and different diseases, but the aggregate of symptoms to be found on careful observation of any well-marked case is sufficiently diagnostic. If the single symptoms, however, cannot be pronounced to be absolutely characteristic there is no room for doubt regarding the nature of the malady when its pathology is investigated. The post-mortem appearances are definite, distinct, and unmistakable, and cannot possibly be confused with anything else in the whole range of human or animal disease. Horse sickness is essentially a disease of the blood and lungs, the most characteristic feature being gelatinous-looking dropsy of the fibrous framework of the lungs, with hemorrhages under the lining of the lung, and a stagnation of greatly-altered blood at definite points in the lung substance, with dropsy in the air cells, these changes being unaccompanied by any inflammation. Appearances in other organs of the body vary according to the duration and severity of the disease, and probably also, in some degree, to the manner in which it has been acquired. At this point the conclusions to be drawn from the symptoms and pathological anatomy are as follows:

That the disease as manifested in the lungs of horses at Port Pirie is in nowise distinguishable from South African horse sickness, which shows such a well-marked aggregation of naked-eye and microscopical appearances as marks it off from all known diseases.

That the disease differs from the most common forms of South African horse sickness in so far as it is more chronic and is associated with less fever and less dropsy of the heart sac and of the pleural cavities. These appearances, dropsy and fever, are what would naturally be expected in the more acute forms of the disease.

The investigation, so far as this State is concerned, might have stopped here, incomplete, in hope that by-and-by investigators elsewhere would work out its history and discover further facts regarding its true cause and cure. But another development in the investigation has changed the whole aspect of the question. On the day on which Mr. Desmond and I made post-mortem examinations of horses in the presence of a number of farmers, horse-owners, and others, Mr. Desmond made a thorough investigation of the bloodvessels of three of the horses, and found aneurisms—(*i. e.*, localized dilatations or swellings) in certain of the smaller arteries of the abdominal cavity, due to the presence of an animal parasite—the *strongylus armatus*. Now, these aneurisms are recognized as not uncommon in horses in some parts of the world, and their dependence on the presence of this parasite is definitely recognized. When very large, they may press upon the diaphragm (midriff), and so cause, to some extent, some of the symptoms one finds in lung sickness; but, in the cases examined, the aneurisms were small, and exerted no pressure whatever on the diaphragm, nor by any mechanical action could they give rise to such symptoms as are commonly associated with their presence in an advanced stage. The problems that now present themselves are:

1. Is the presence of the *strongylus* in those particular horses a mere accidental occurrence, having no reference whatever to the lung sickness?

2. Is it possible that the *strongylus*, in a way not hitherto understood and not as yet recognized, may cause all the symptoms that appear in this South Australian horse sickness?

3. Is the horse sickness in South Africa associated with, or caused in any way by, the presence of *strongylus* in the abdominal arteries?

4. Are we dealing with a form of lung sickness that may have two distinct and unconnected causes—a fungus in South Africa and an animal parasite in South Australia?

These are problems that demand solution, not in the interest of pathological science so much as in the interests of State economies and of the particular individuals concerned.

A horse belonging to Mr. Crispin, which had been suffering from the symptoms of horse sickness for some months, died and was subjected to a post-mortem examination by Mr. Desmond. The lungs presented the appearances described as characteristic of the disease, and there were numerous aneurisms, associated with *strongyli*, in the branches of the abdominal aorta.

It may be well to mention that a horse in another part of the State suffered from symptoms of lung sickness, and was found post-mortem to show the lung characteristics and aneurisms with *strongyli* in the same way as the Port Pirie horses.

---

Drs. Buckley, Walters and Swan, of the Veterinary Department of Street Cleaning, of the city of New York, have given it as their professional opinion that the mallein test for glanders of horses is unreliable and worthless. Surely this is not in accord with the experience at home and abroad in the use of this product as a diagnostic agent.

